# Altered mean platelet volume in patients with polymyositis and its association with disease severity

**DOI:** 10.1590/1414-431X20165168

**Published:** 2016-05-13

**Authors:** Y.-F. Peng, Y.-X. Huang, Y.-S. Wei

**Affiliations:** Department of Laboratory Medicine, Affiliated Hospital of Youjiang Medical University for Nationalities, Baise, Guangxi, China

**Keywords:** Mean platelet volume, Polymyositis, Disease severity

## Abstract

Polymyositis (PM) is an autoimmune disease characterized by chronic inflammation in skeletal muscle. Mean platelet volume (MPV), a marker in the assessment of systemic inflammation, is easily measured by automatic blood count equipment. However, to our knowledge, there are no data in the literature with respect to MPV levels in PM patients. Therefore, in this study we aimed to investigate MPV levels in patients with PM. This study included 92 newly diagnosed PM patients and 100 healthy individuals. MPV levels were found to be significantly lower compared with healthy controls (10.3±1.23 *vs* 11.5±0.74 fL, P<0.001). Interestingly, MPV was found to be positively correlated with manual muscle test (MMT) score and negatively correlated with erythrocyte sedimentation rate (ESR) in patients with PM (r=0.239, P=0.022; r=−0.268, P=0.010, respectively). In addition, MPV was significantly lower in active PM patients compared with inactive PM patients (9.9±1.39 *vs* 10.6±0.92 fL, P=0.010). MPV was independently associated with PM in multivariate regression analyses, when controlling for hemoglobin and ESR (OR=0.312, P=0.031, 95%CI=0.108 to 0.899). The ROC curve analysis for MPV in estimating PM patients resulted in an area under the curve of 0.800, with sensitivity of 75.0% and specificity of 67.4%. Our results suggest that MPV is inversely correlated with disease activity in patients with PM. MPV might be a useful tool for rapid assessment of disease severity in PM patients.

## Introduction

Mean platelet volume (MPV) is determined by megakaryocytes during platelet production, and is associated with platelet activation and function (1). Elevated MPV indicates both increased platelet volume and number of large-sized platelets. Several lines of evidence attest that increased MPV is associated with fibromyalgia, myocardial infarction and cerebrovascular disease (2–4). In contrast, decreased MPV has been observed in some rheumatologic diseases such as ulcerative colitis, ankylosing spondylitis and rheumatoid arthritis (5 – 7). In fact, MPV has been regarded as an inflammatory index in various diseases (8).

Polymyositis (PM) is an autoimmune disease characterized by chronic inflammation of skeletal muscle (9). Accumulating data demonstrates that increased interleukin (IL)-1, (IL-6) and tumor necrosis factor (TNF) are correlated with PM and are indicators of inflammatory burden in patients with PM (10,11). Previous studies show that C-reactive protein (CRP) and erythrocyte sedimentation rate (ESR) are elevated in patients with PM compared with healthy controls (12). Very recently, a strong relationship between serum hyaluronic acid and PM has been reported by Silva et al. (13), which suggests that inflammatory cytokines are primarily responsible in the pathogenesis of PM patients. MPV, a marker in the assessment of systemic inflammation, is easy to be measured by automatic blood count equipment. However, to our knowledge, there is no investigation in the literature with respect to MPV levels in PM patients. Therefore, in this study, we aimed to investigate MPV levels in patients with PM.

## Patients and Methods

### Patients

This study included 92 newly diagnosed PM patients attending the Affiliated Hospital of Youjiang Medical University for Nationalities, Baise, Guangxi, China, who fulfilled the Bohan and Peter criteria (14). Clinical and laboratory data of patients were obtained from medical records. Patients with other systemic autoimmune diseases, cancer-associated myositis, hypertension, diabetes, hematological disorders, chronic renal or hepatic insufficiencies, cardiovascular disease, acute or chronic infectious diseases, thrombotic disease, malignant tumors, mental disorders, and pregnancy were excluded from the study. After a detailed medical history and physical examination, a total of 100 healthy individuals undergoing routine physical examinations in our hospital were included as healthy controls. Disease activity was estimated by using manual muscle test (MMT) score in patients with PM, which indicates muscle strength (15). Neutrophil count, lymphocyte count, hemoglobin, platelet count, CRP, ESR and MPV levels of patients with PM were retrospectively collected from medical records. Complete blood count was performed using an automated hematology analyzer (Sysmex XN2000, Japan).

The study was approved by the Affiliated Hospital of Youjiang Medical University for Nationalities institutional review board, and all participants provided written informed consent.

### Statistical analyses

Data were analyzed using SPSS16.0 statistical software (IBM, USA). Distribution of data was assessed by Kolmogorov-Smirnov test. Differences between numeric variables were tested with Student's *t*-test or Mann-Whitney U-test. The differences in proportions between groups were compared with the chi-square test. Correlation analysis was performed with the Spearman approach. Multivariate logistic regression analysis was used to identify independent parameters associated with PM. The ability of MPV to predict disease activity was evaluated using receiver operating characteristic (ROC) curve analysis. All P values were two-sided and a value of <0.05 was considered to be statistically significant.

## Results

**Figure 1:**
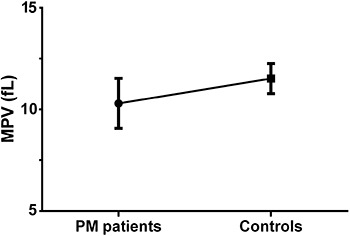
Mean platelet volume (MPV) in polymyositis (PM) patients and healthy controls. P<0.001, Student's *t*-test.

Demographic characteristics and laboratory data of all individuals are reported in Table 1. There were significant differences between PM patients and healthy controls in terms of gender, CRP, ESR, hemoglobin, and lymphocyte and neutrophil count. Of note, MPV levels were found to be significantly lower compared with healthy controls (10.3±1.23 *vs* 11.5±0.74 fL, P<0.001), as shown in Figure 1.

**Table:**
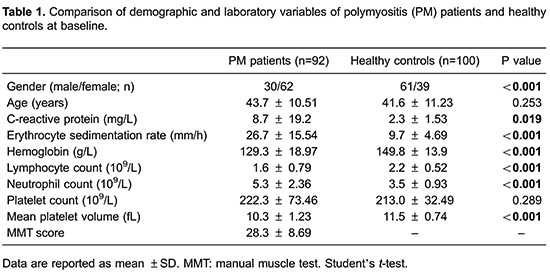

**Figure 2:**
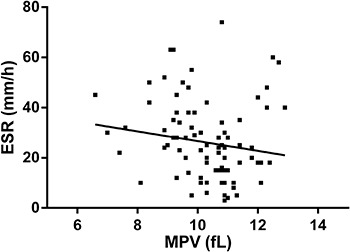
Correlation between mean platelet volume (MPV) and erythrocyte sedimentation rate (ESR) in patients with polymyositis (PM).

**Figure 3:**
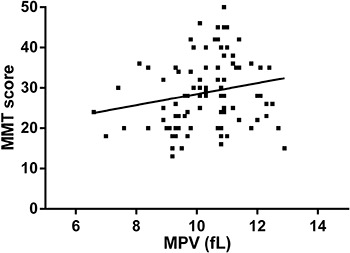
Correlation between mean platelet volume (MPV) and manual muscle test (MMT) score in patients with polymyositis (PM).

The results of the correlation analysis between MPV and laboratory findings revealed that MPV was negatively correlated with platelet and neutrophil counts in PM patients (r=−0.500, P<0.001; r=−0.540, P<0.001, respectively). Interestingly, MPV was found to be positively correlated with MMT scores and negatively correlated with ESR in patients with PM (r=0.239, P=0.022; r=−0.268, P=0.010, respectively; Figures 2 and 3). In addition, MPV was significantly lower in active PM patients compared with inactive PM patients (9.9±1.39 *vs* 10.6±0.92 fL, P=0.010), as shown in Table 2.

**Table:**
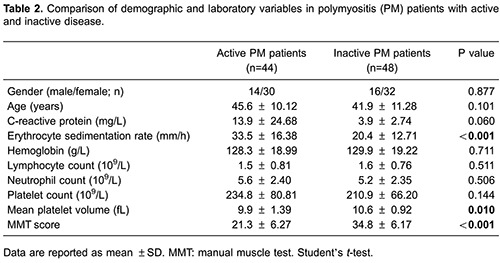

**Figure 4:**
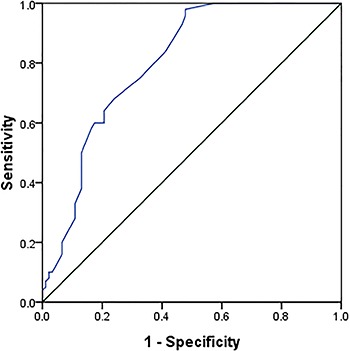
Receiver operating characteristics (ROC) curve analysis for mean platelet volume (MPV) in patients with polymyositis.

After adjusting for demographic characteristics, hematologic parameters, and inflammatory indicators (gender, CRP, ESR, hemoglobin, lymphocyte count and neutrophil count), MPV was associated with PM in multivariate regression analyses (OR=0.312, P=0.031, 95%CI=0.108 to 0.899) (Table 3). The ROC curve for MPV in estimating PM patients was constructed, and the area under the curve of 0.80 was found (95%CI=0.736 to 0.864, P<0.001; Figure 4). The cut-off values of MPV were 10.85 fL with sensitivity of 75.0% and specificity of 67.4%.

**Table:**
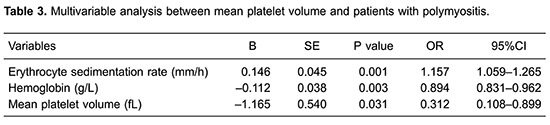

## Discussion

To the best of our knowledge, this is the first study to reveal the potential clinical value of MPV in PM patients. In the present study, we demonstrated that the levels of MPV were lower, and presented a trend to correlate with disease severity in patients with PM.

Complete blood count test is a routine examination in the diagnosis and follow-up period of rheumatoid disease, and MPV is one of the test's components. Increased MPV has been considered to be a marker of thrombocyte activation, and has been found to have a pivotal role in the pathogenesis of cardiovascular disease (16). Moreover, a correlation between MPV and acute phase reactants was observed in rheumatoid arthritis (17). Increased MPV has been associated with preeclampsia, varicocele, chronic embolism pulmonary hypertension and pulmonary embolism (18 [Bibr B19] – 21). It has been shown that MPV is increased in patients with acromegaly, juvenile idiopathic arthritis and proteinuria (22–24). However, Kapsoritakis et al. (25) reported an association between decreased MPV and Crohn's disease, suggesting that MPV is a useful marker of inflammatory bowel disease activity. These observations indicate that MPV could be used in the evaluation of some inflammatory disorders.

PM is an idiopathic inflammatory myopathy with systemic inflammation. There is evidence that IL-6 is increased and positively correlated with CRP in patients with PM (26). Several other inflammatory cytokines, such as IL-4, IL-8 and TNF, have also been reported to be increased in PM patients (11). In fact, the hematopoietic functions in the body are presumably mediated and influenced by these inflammatory cytokines (27). Indeed, these cytokines are responsible for inflammation and have various effects on hematopoiesis in some inflammatory disorders (11). On the other hand, large-sized platelets are more frequently found as a result of a higher concentration >of inflammatory substances (27). A negative correlation between MPV and platelet counts in some pathological conditions indicates a tendency to maintain hemostasis by preserving a constant platelet mass (28). This negative relationship is frequently observed in inflammatory disorders, in which reactive large-sized platelets migrate to inflammatory sites where these platelets are massively consumed (29,30). Likewise, high-grade inflammation leads to a decrease in MPV in some rheumatoid diseases, also possibly due to the increased consumption of large-sized platelets at the inflammation site (30). Therefore, a reasonable explanation for the low levels of MPV in PM patients would be that high-grade inflammatory states in muscle tissue of PM patients may increase the consumption of large platelets.

The current study, however, has several limitations. First, because PM is a relatively rare disease a limited number of cases were included. The retrospective study design is also not ideal. In addition, the levels of MPV were not evaluated in PM patients undergoing required anti-inflammatory medication. However, our results suggest that lower MPV is correlated with disease activity in patients with PM, and therefore, MPV may be useful for a rapid assessment of disease severity in PM patients.
